# Environmental Implications Associated with the Development of Nanotechnology: From Synthesis to Disposal

**DOI:** 10.3390/nano12234319

**Published:** 2022-12-05

**Authors:** Otávio Augusto L. dos Santos, Bianca Pizzorno Backx, Rasha A. Abumousa, Mohamed Bououdina

**Affiliations:** 1 Instituto de Bioquímica Médica, Universidade Federal do Rio de Janeiro, Rio de Janeiro 21941-902, Brazil; 2 Campus Duque de Caxias, Universidade Federal do Rio de Janeiro, Duque de Caxias 25240-005, Brazil; 3 Department of Mathematics and Science, Faculty of Humanities and Sciences, Prince Sultan University, Riyadh 11586, Saudi Arabia

**Keywords:** nanomaterials, green synthesis, environment, risks, toxicity, transformations

## Abstract

Nanotechnology remains under continuous development. The unique, fascinating, and tunable properties of nanomaterials make them interesting for diverse applications in different fields such as medicine, agriculture, and remediation. However, knowledge about the risks associated with nanomaterials is still poorly known and presents variable results. Furthermore, the interaction of nanomaterials with biological systems and the environment still needs to be clarified. Moreover, some issues such as toxicity, bioaccumulation, and physicochemical transformations are found to be dependent on several factors such as size, capping agent, and shape, making the comparisons even more complex. This review presents a comprehensive discussion about the consequences of the use and development of nanomaterials regarding their potential risks to the environment as well as human and animal health. For this purpose, we reviewed the entire production chain from manufacturing, product development, applications, and even product disposal to raise the important implications at each stage. In addition, we present the recent developments in terms of risk management and the recycling of nanomaterials. Furthermore, the advances and limitations in the legislation and characterization of nanomaterials are also discussed.

## 1. Introduction

Nanotechnology involves materials at a scale ranging from 1 to 100 nm in one of its dimensions. It has brought advances in several areas such as electronics, pharmaceuticals, biotechnology, agriculture, cosmetics, and food [1,2,3,4,5,6,7,8]. Nanostructures have a higher surface-to-volume ratio compared to bulk materials aside from exhibiting enhanced catalytic, mechanical, optical, electrical, tribological, thermal, and other properties [9]. For this reason, nanomaterials have been widely studied and applied for the production of different products such as textiles, food coloring, pharmaceuticals, and cosmetics [9]. Given this demand for nanomaterials, their exposure becomes inevitable and must be considered, needing further discussions about the risks to health and the environment.

The characteristics of NMs such as size, shape, surface area, stability, and the coating compounds’ intrinsic properties are essential to assess the potential risks [10]. These factors will influence the pathway nanomaterials to interact with biological systems and the possibility of causing harm [11]. Unfortunately, there is still limited information in the literature about the impact of nanostructured particles on the health of living beings and the environment. In addition, we must evaluate the toxicity of the final products, the entire production chain of nanomaterials, and all the products generated from their interaction with complex systems.

**Figure 1:**
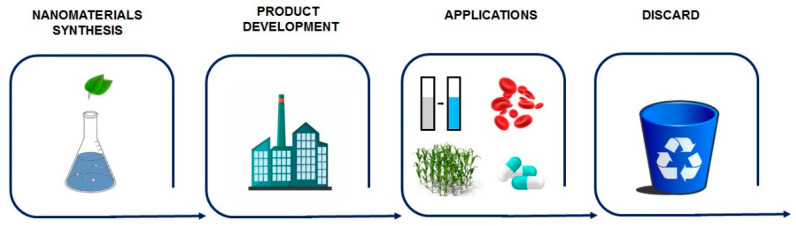
The life cycle of NMs. It begins with extensive research and development of an appropriate synthesis route, followed by the development of a product and its consequent use. Finally, it is necessary to correctly dispose of the NM products when no longer utilized.

This review presents different aspects involving nanomaterials regarding their potential risks considering the entire production chain, starting from the manufacturing process, product development, applications, and disposal (Figure 1). Knowing the risks at each stage and the associated factors can be crucial in searching for alternatives to minimize such problems and develop more sustainable approaches. In addition, we present the advances and challenges in the legislation and regulation of nanomaterials.

## 2. Synthesis of Nanomaterials

Top–down and bottom–up methodologies have been adopted to synthesize NMs through different routes such as physical, chemical, and biological. Physical and chemical methods are widely used. However, they usually require toxic solvents and generate by-products that have high-risks to the environment and harm human and animal health, hence limited applications [12,13].

Recently, the biogenic approach has gained notoriety. In this synthesis method, biological systems or their products are used in the process. Microorganisms, plant extracts, or isolated biomolecules such as sugars, nucleic acids, and proteins are being investigated for this purpose [14,15,16,17,18,19,20,21,22,23,24,25,26]. It has no or low toxicity besides being an inexpensive and straightforward methodology [21,27,28].

Green chemistry aims to produce nanomaterials sustainably to reduce the maximum waste generated, energy consumption, and the concentration of precursors required. It replaces toxic compounds and solvents with benign or less toxic alternatives such as plant extracts. Another example consists of the use of water instead of organic solvents. Furthermore, reducing the steps during the synthesis process will spare wasting reagents and energy. Another essential factor to be considered is that more eco-friendly conditions of pH, temperature, and pressure are adopted during green synthesis and the establishment of protocols with reactions that generate few or no by-products. Thus, the synthesis needs to be sustainable throughout the process including the disposal [29,30,31,32].

One of the most studied routes is using plant extracts as reducing agents and surface stabilizers. Different plant parts have already been used such as fruits [33,34,35,36], roots [26,37,38], peels [24,39], seeds [40,41], leaves [22,42,43,44], and flowers [45]. In a bottom–up method, the nanostructure control can be achieved through multiple factors such as pressure, temperature, and the concentration of reagents, among others [46]. The synthesis process starts with a metal precursor, usually an inorganic salt. In a reaction medium, this salt suffers an ionic dissociation and biomolecules present such as proteins, sugars, flavonoids, and tannins could act to reduce these ions to a reduced state, assuming the state of its fundamental atom in a nucleation phase where a stable nucleus is formed by reducing the metallic precursor. Subsequently, the interaction with other nuclei begins to aggregate, followed by an increase in nuclei size during the growth phase. This phase can be interrupted by depleting the metallic precursor or coating molecules to prevent the aggregation of these nanostructures [27,47,48,49]. Agents capable of stabilizing nanostructures are essential for the formation of a colloidal solution without aggregates [50]. This occurs because the lack of bonds on the surface increases the Gibbs’ free energy, making this highly reactive and the nanostructure very unstable. As a result, it has a high contact surface area compared to its volume. In this way, due to dissatisfied bonds, the network parameter of the structure is reduced. Consequently, the atoms are closer together, changing the material’s properties as a whole [51]. Due to the Gibbs’ high energy, nanostructures tend to clump together to form larger particles. The synthesis process and reagents involved will directly influence the morphology and size of the resulting nanomaterial [50].

## 3. Applications

A variety of products containing NMs are already on the market and under development. However, through their production and utilization, these NMs can reach the environment and may cause contamination and pollution. Furthermore, how NMs are used interferes with the nature of risk exposure. Below, some of the most common applications of NMs and their main contamination targets will be presented and discussed (Table 1). A review published in 2015 estimated that until October 2013, 1814 consumer products from 622 companies in 32 countries owned NMs. The health and fitness category itself accounts for about 42% of the total products. Nanosilver is the most frequent (24%); however, 49% of the products (889) do not provide the chemical composition of the used NMs and the majority (71%) of the products do not present enough supporting information to corroborate the claim of the used NMs [52].

One of NM applications is cosmetics, mainly in sunscreens. TiO_2_ and ZnONPs are used for their absorbing properties of ultraviolet (UV) radiation and hypoallergenicity. To constitute a transparent emulsion, they must have nanometric dimensions [11,12]. Another application is nanoemulsions, which use NPs to achieve textures, optical, and tactile characteristics specific to each product [12]. The main effect of the possible risks of these products would be on the aquatic environment and living beings in that environment, and direct contact with the skin when using such products [12]. However, many of these products have been extensively tested for health and safety by regulatory authorities. As an example, ZnONPs are already incorporated in sunscreens and lip balms [11]. 

Nanotechnology has also emerged as a potential alternative for environmental decontamination. Various nanomaterials such as NPs, carbon nanotubes (CNTs), graphene, and nanocomposites have been extensively investigated for contaminant remediation [1]. Studies have shown promising results on removing environmental pollutants by NPs, making room for future use in ecological disasters like oil spills and other environmental contaminants such as heavy metals [89,90,91]. TiO_2_ NPs have been investigated for their photocatalytic properties to eliminate organic compounds. In this context, gold nanoparticle decorated TiO_2_ (Au/TiO_2_) produced using green tea exhibited an adsorption capacity greater than 8185 mg/g of methylene blue [65]. Carbon nanomaterials have been investigated as an alternative for treating organic contaminants in soil and water. This ability is related to surface hydrophobicity and the greater affinity of these NMs to these compounds than common particles in natural environments [1]. Hydroxyl-functionalized multi-walled carbon nanotubes (MWCNT-OH) showed the ability to remove naphthalene and fluorene from water bodies [62]. In another study, CNTs have shown the ability to adsorb four polycyclic aromatic hydrocarbons (naphthalene, fluorene, phenanthrene, and pyrene) from the soil, respectively [92]. For this application, NMs are generally used for fluid media treatment associated with support such as polymers, glass, and metals. While for soil treatment, they are added directly to the contaminant. Once adhered to a matrix during the decontamination process, rain or effluents can carry these NMs to the aquatic environment [12]. Additionally, when applied directly to the soil, the effects on organisms present such as plants, insects, and the terrestrial microbiota must be considered. 

Furthermore, many studies have reported the antimicrobial activity of different nanostructures such as silver, gold, copper, and titanium NPs [3,53,54]. These specific and unique properties make them very promising for diverse applications such as in medical-hospital products, clothing, air purifiers, and food packaging, in which AgNPs stand out among the rest [3,46,47,93]. In general, the NMs showed microbicidal activity, even in lower concentrations, below a concentration sufficient to cause harm to animals, and these effects were found to be a dose-dependent [33,94,95,96,97]. In addition, it was reported that NMs produced by green synthesis could be less toxic and did not show significant cellular toxicity at the concentrations tested in many studies [98,99]. In this context, it is possible to study a safe range of NM concentrations for their use without causing any health concerns and implications. Their use has been tested in catheters, bone prostheses, cardiovascular implants, and hospital textiles [24,26,100]. The main risk to the environment arises from the disposal of these NMs, whereas the health risk originates from their direct administration.

In addition, NMs have offered new approaches for diagnosing diseases, imaging, administration and drug formulations as well as therapy, particularly those with antimicrobial and antitumor potential. Some NPs have demonstrated that they can be used to selectively combat tumor cells without causing significant effects on normal cells [69,70,71,72,73,74,75]. This selectivity may be associated with pH, since the stability of NMs is pH dependent, and tumor cells create a microenvironment that is slightly more acidic than normal cells, which could cause the dissolution of NPs [71]. The antitumor effect has also been associated with the fact that tumor cells have high cell metabolism and rapid cell division [74]. NMs with photocatalytic properties can also be used to control drug release, sensitize cells, or even create an oxidative environment leading to cell death [76].

NMs can also be used for drug delivery, enabling them to deliver drugs with greater efficiency, reducing toxicity, and enhancing their effects. For example, oleic acid (OA)-pluronic-coated iron oxide magnetic NPs were efficiently associated with the antitumor agent doxorubicin. This association allows for sustained drug release, and aside from the cytotoxic effect on tumor cells, was found to be dose-dependent [66]. Similarly, AuNPs functionalized with a thiolated poly(ethylene glycol) (PEG) and conjugated to the antitumor drug oxaliplatin demonstrated an as good as or significantly better antitumoral activity against lung and colon cancer cells than oxaliplatin alone [67]. The anti-inflammatory potential has also been reported for different NMs such as cerium oxide NPs (CeNPs) [101], AgNPs [33,102,103], AuNPs [104,105], ZnONPs [106,107], SeNPs [108], and PtNPs [109]. In recent years, NMs have also been investigated for their potential in regenerative medicine. They can be applied for the control of the agents’ release, modification of the characteristics such as solubility and stability of molecules and help control the properties of scaffolds that serve as the support for cells [110]. 

In addition, the possibility of using nanotechnology in agriculture has attracted great attention. NMs can be used to increase agricultural productivity, reduce costs, and improve crop management efficiency [111]. Nanoformulations and NPs have been investigated as a vehicle for delivering pesticides to decrease their toxicity, increase stability and action, or directly combat pests and diseases [86]. Furthermore, sensors can be used to detect the presence of pathogens and pesticides and monitor plantation and environmental conditions such as air and soil, allowing for precision agriculture [86]. Fertilizers have also been studied to efficiently handle inputs, with minimal loss and a reduction in environmental contamination [86,87,88]. However, it is essential to investigate how these NMs accumulate and degrade in the environment and their toxicity to terrestrial and aquatic environments.

Finally, nanotechnology has allowed for the development of nanosensors capable of detecting a variety of target analytes, which can be molecules, nucleic acids, proteins, and even cells such as bacteria [60,112]. Nanosensors can be divided into optical, electrochemical, and magnetic, and applied in various processes such as in the diagnosis of diseases either in vivo or in vitro in the detection of pollutants in environmental samples, monitoring the concentration of molecules of interest in real-time, monitoring environmental conditions such as humidity and temperature, or in any process that needs to detect or monitor the presence of a target analyte [112]. The development of nanosensors for glucose, H_2_O_2_, uric acid, urea, neurotransmitters, hormones, drugs, metals, pesticides, viruses, bacteria, and even tumors has already been widely reported in the literature [58,59,60,61].

## 4. Environment Transformations

**Figure 2:**
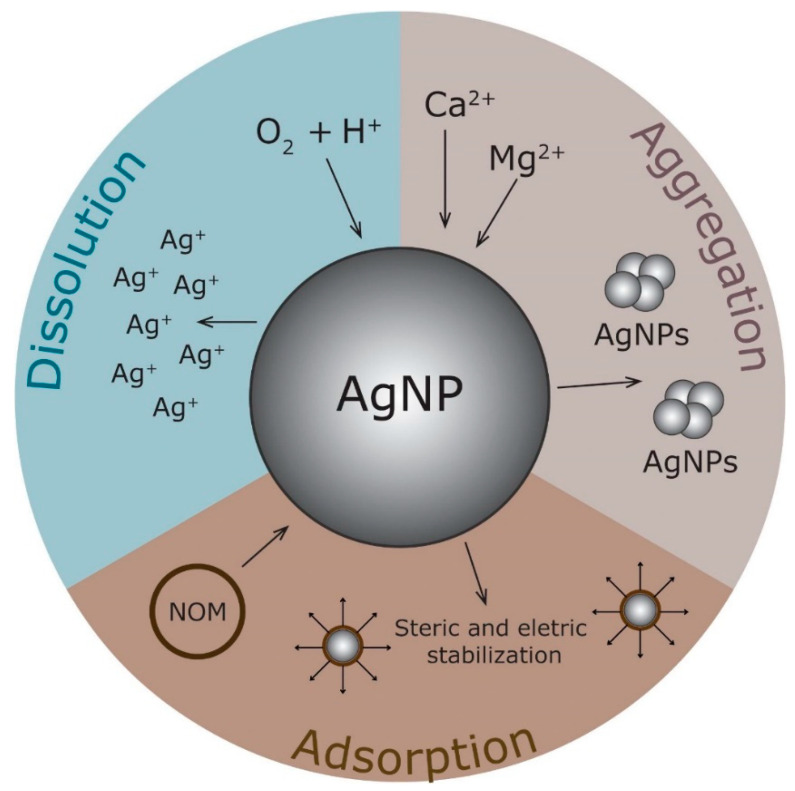
NM transformations in the environment. Depending on the environment they are in, nanomaterials can undergo physical-chemical transformations, involving aggregation, dissolution into its ions, or the adsorption of molecules on their surface as natural organic matter (NOM). Reprinted with permission from Jorge de Souza, Rosa Souza and Franchi, (2019). Copyright © 2019 Elsevier Inc.

A multifactorial assessment is needed to understand the possible harmful effects of NMs. In this context, it is important to highlight that the properties of NMs vary considerably depending on the particle size, shape, chemical composition, capping agent, charge, solubility, and aggregation state [113]. NMs usually have their surface coated by chemical functional groups to guarantee their colloidal stability including proteins, polymers, carboxylic acids, surfactants, and sugars [100]. Nevertheless, these properties suffer interference from the medium, thus requiring the characterization of NPs in the means of production used in toxicological tests and the environment. However, nanomaterials suffer interference from the environment in which they are, and their properties and characteristics can change. Thus, it is necessary to consider these possible transformations for a more accurate toxicity evaluation. Variations in pH, the stability of ionic strength, and the concentrations and the type of organic compounds are also found to influence the NMs’ behavior, bioavailability, and toxicity. In addition to the above critical factors, to better assess the real risks in-depth and breadth, one must consider how NMs will present themselves after interacting with the environment, whether in a cellular, terrestrial, or aquatic environment [12,113]. In general, it can be possible to divide this transformation after interaction into three distinct events: adsorption, aggregation, or dissolution, as shown in Figure 2 with silver nanoparticles as an example.

When in contact with the medium, NMs can adsorb components onto their surface. This affinity can originate mainly from electrostatic interactions, hydrogen, and hydrophobic bonds [1]. The adsorption process and mechanism mainly depend on the particle size, shape, surface charge, and the capping agent [114]. For example, if the capping agent interaction with the NM surface is weak, it can be replaced by other ligands with a stronger affinity [115]. In this context, the AgNPs with a size of 65 nm and spherical shape presented a zeta potential close to 0 and showed a tendency to agglomerate in the water. The addition of bacterial exopolysaccharides (EPS) to the medium increased the negative surface charge of NPs to values greater than −30 mV, and their aggregation was reduced. In addition, the adsorption of the polysaccharide onto the surface increased the stability of NPs and the EPS adsorption was found to be dependent on pH as well as the salt and EPS concentrations [116].

**Figure 3:**
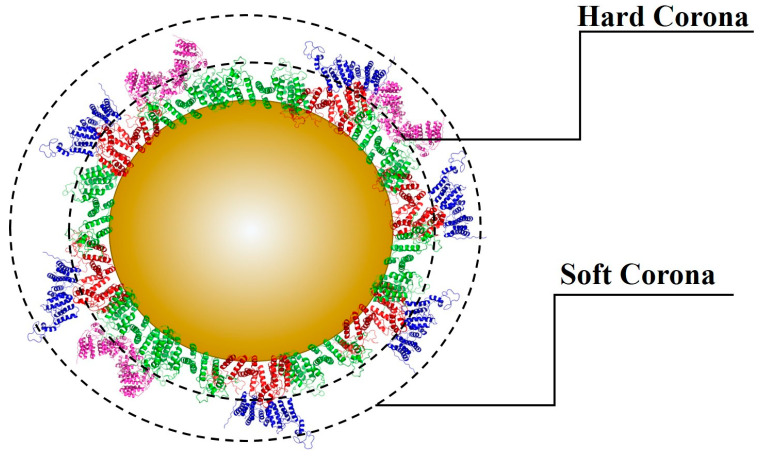
Protein corona consists of two layers: the hard corona is an inner layer composed of high-affinity proteins, and the soft corona is an outer layer that is weakly bound by low-affinity proteins not directly bound to the nanomaterial.

Especially in biological media, NMs can be rapidly coated with proteins to form a protein corona. The latter consists of two layers (Figure 3): the hard corona is an inner layer composed of high-affinity proteins while the soft corona is an outer layer that is weakly bound by low-affinity proteins not directly bound to NPs [114,117]. Proteins can interact at the surfaces of NMs by noncovalent interactions and chelate with oxidized metals [118]. This protein corona is dynamic and changes composition over time due to the affinity-based competition between proteins and other molecules like organic matter for adsorption onto the surfaces of the NPs [114,118].

The composition of the biological environment can affect the dynamic protein exchange processes between the environment and the protein corona, thereby influencing the properties of the protein corona. However, NMs can adsorb many different molecules at the surface including polysaccharides, proteins, lipids, nucleic acids, metabolites, and other molecules, named the Eco-corona. In this case, protein may not be the most abundant constituent, mainly if it is formed outside an organism [118]. The protein adsorbed onto the NM surface can alter all of the interaction dynamics. It can affect the protein structure and lead to an immune response [114]. It can block the attachment of ligands to the surface of the NPs or interfere with the interactions between the ligands and receptors, and therefore alter subsequent phagocytosis and signaling transduction in cells. The adsorption of proteins could lead to various modifications in the physicochemical characteristics of NMs including the size, shape, surface chemistry, stability, biocompatibility, and composition, as a consequence, potentially contributing to changes in the NP uptake [114,117]. As an example, the protein corona increases the stability of particles, and prevents dissolution and ion release in ZnONPs [119]. Similarly, the presence of four serum proteins, immunoglobulin G, fibrinogen, apolipoprotein A1, and human serum albumin enhanced the AuNPs stability at pH 7.4 (physiological) and pH 6.2 with high ionic concentration (tumoral microenvironment) [120]. However, some negative effects have been also observed. For example, titanium dioxide (TiO_2_) NPs were found to induce conformational changes of tubulin, thus inhibiting its polymerization properties [121]. It has also been reported that AuNPs could induce changes in the conformation of bovine serum albumin (BSA) [122] and fibrinogen, whereby such changes were able to activate an inflammatory response [123].

The dissolution process is related to the transformation of NMs such as NPs into their ion form. These ions’ release of NMs is associated with higher toxicity [1,113]. In biological media, these ions can bind to proteins and other macromolecules or precipitate as insoluble salts such as AgCl or Ag_3_PO_4_. For example, chloride ions can interact with Ag ions to form AgCl, precipitating in the solution. As a result, fewer Ag ions are available, and the toxicity is reduced [124]. Some reports have evaluated the dissolution kinetics of NPs under different conditions [125,126,127]. Liu et al. (2010) observed that dissolved oxygen and protons are essential for releasing ions through the oxidation process in a liquid medium while the Ag^+^ release from the ions increases under high temperatures and decreases in alkaline pH. The addition of organic matter such as humic or fulvic acids can also decrease the dissolution rate of AgNPs.

**Figure 4:**
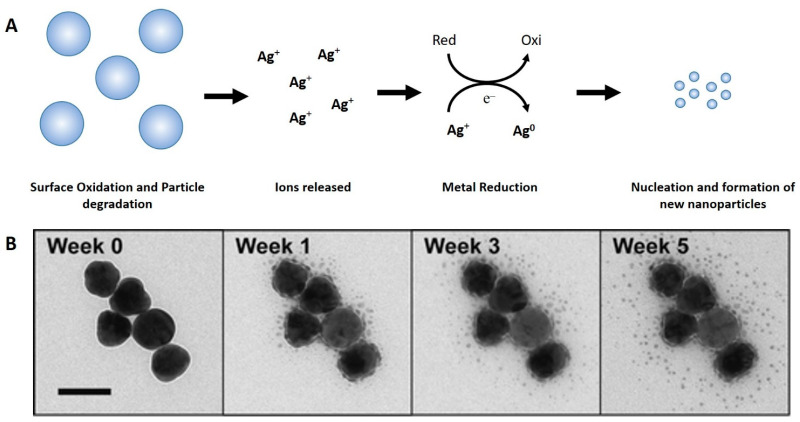
(**A**) Proposed pathway for new particle formation by the dissolution process of the parentals. First, surface oxidation occurs with ambient oxygen and water. Ionized silver diffuses away from the parent particle in the water layer driven by the concentration gradient. New particle formation by nucleation via the chemical and/or photochemical reduction of silver ions. (**B**) Formation of small NPs in the vicinity of 75 nm AgNPs at 100% relative humidity over five weeks. TEM images during the exposure period show increasing numbers of small NPs throughout this period (Glover, Miller and Hutchison, 2011). Reprinted with permission from Glover, Miller and Hutchison, (2011). Copyright © 2011, American Chemical Society.

Moreover, the formation of new NMs resulting from the dissolution of another has already been proposed (Figure 4). In environments with a humidity greater than 50%, new NPs formed close to their parents. This phenomenon is directly dependent on humidity and has been observed for different NPs with different coatings. This process occurs in three steps: first, the oxidation and dissolution of the nanoparticle surface occur in the presence of water, releasing its ions; second, these free ions in aqueous media can undergo chemical or photoreduction, forming an elemental state; third, this metal in the elemental state gives rise to nuclei that interact and form new smaller NPs. Nanoparticles stored at 0% humidity remained unchanged, but some stored under humid conditions showed morphological transformations such as changes in the shape, size, and the total number of NPs. These effects could even be observed within a few hours [128]. 

Furthermore, NMs can interact with each other and aggregate without a stabilizing coating, or when the repulsive force is weaker than the attractive force [113]. In addition, ionic strength is also an important factor in aggregation, mainly in acid pH. A higher concentration of positive charges such as Ca^2+^ and Mg^2+^ can affect the NMs’ surface charge, removing the repulsive energy, and leading to aggregation [115]. The ionic strength of freshwater environments can vary from approximately 1 to 10 mM and is around 700 mM for saline environments. Therefore, NMs tend to be unstable in these environments [124,129]. The correlation of pH and surface charge is another critical factor in NP aggregation. At the pH corresponding to the point zero charge, NMs become unstable and aggregate [115]. Larger and aggregated NMs have less surface area, thus the lower toxicological potential. However, aggregates can carry toxic contaminants [113].

To expand the understanding of these transformations, AgNPs were studied in four different aquatic microcosms: surface water; water and sediment; water and aquatic plants; or water, sediment, and aquatic plants. Polyvinylpyrrolidone (PVP) or gum Arabic (GA) coated AgNPs were introduced into these environments. In response to the presence of AgNPs, plants released dissolved organic matter (DOM) into the water to bind Ag ions and decreased their toxicity. The plant-derived DOM had a stabilizing effect on the PVP-AgNPs but led to the removal of the GA coating from the GA-AgNPs and dissolution. These released ions bind to sediments or plant surfaces associated with thiol groups and present as oxides. In microenvironments without plants, PVP-AgNPs aggregated extensively, which was not observed for GA-AgNPs [130]. 

Natural colloids in river water were the main ones responsible for causing the aggregation of CeO_2_ NPs. At concentrations lower than 1 mg/L, colloids in the samples led to heteroaggregates, followed by sedimentation. However, at higher concentrations (10 mg/L and 100 mg/L), homoaggregation was more important than interaction with natural colloids as being mainly responsible for aggregation. When the water samples were filtered, almost no sedimentation was observed, and the CeO_2_ NPs were more stable. The stability of the NPs was directly related to the initial concentration and the presence of colloids, which may be due to the increased frequency of collisions, consequently causing aggregation and sedimentation [131]. In another study, the stability of CuONPs was studied against the pH change, increasing the ionic and valence strength, and natural organic matter (NOM). The particle’s hydrodynamic diameter increased from 240 nm to 270 nm in alkaline pH, while at pH 3, the diameter reduced to 124.68 nm. Additionally, at pH 3, the zeta potential was close +20 mV and at pH 9, it was near −25 mV. Thus, the aggregation was reduced with the increase in the electrostatic repulsive force. In addition, higher ionic and valence intensified the aggregation and sedimentation of the CuONPs due to the compression of electrical double layers. At 100 mM NaCl, the hydrodynamic size increased, further reaching 508 nm, which indicates that the divalent electrolyte further reduced the stability of NP suspension and promoted their aggregation. The presence of humic acid and citric acid enhanced the dispersion and stabilization of CuONPs. However, L-cysteine caused a significant increase in the hydrodynamic size, varying considerably from 324 up to 825 nm. The soluble Cu released from CuONPs (100 mg/L) was up to 56.25% of the total Cu at pH 3. Still, it was only 0.19% at pH 7, showing a negative correlation between the solution pH and CuONPs solubility. NaCl significantly promoted the dissolution of CuONPs in 100 mM NaCl solution reaching five times more than that in control. However, compared to NaCl at the same ionic strength, CaCl_2_ with a higher ionic valence showed no marked effect on the dissolution of CuONPs at low ionic strength (10 mM) but slightly inhibited the release of Cu^2+^ at much higher ionic strength (100 mM). The result suggests that the ionic strength and the type of electrolyte impacted the release of CuONPs. In the absence of NOM, the nominally dissolved Cu concentration in the CuONPs suspension was only 0.16 mg/L. While in the presence of 100 mg/L NOM, the concentration of Cu was increased to 2.80 ± 0.14 mg/L for humic acid, 19.48 ± 0.41 mg/L for citric acid, and 1.73 ± 0.52 mg/L for L-cysteine, suggesting a strong effect of NOM, especially citric acid, on promoting the soluble Cu released from CuONPs. Thus, pH and NOM are critical factors in the dissolution of CuONPs, with more soluble Cu occurring at lower pH and higher NOM [132]. The AgNPs’ stability was studied in the presence of different halide ions. NaF induced the aggregation only at concentrations above 75 mM, while NaCl induced it largely. For NaI or NaBr, the aggregation was less pronounced. This can explain because AgF’s solubility is much higher than other halides [133].

## 5. Toxicity

### 5.1. Aquatic Environments

In aquatic environments, NMs are present in free form or interact with organic and inorganic ligands. Meanwhile, their speciation is influenced by the physical and chemical properties of the environment. The aquatic environment is the most studied since it is the primary recipient of contaminants. Once NMs contaminate the aquatic environment, ingestion through water may occur. However, the mechanisms that living organisms respond to intoxication caused by NMs are still not well understood. An early study was devoted to assessing marine invertebrates’ toxicity toward TiO_2_ and C_60_ NPs. *Daphnia* organisms are bioindicators because they interact with large portions of water in the environment. Therefore, polluting NPs’ ingestion has tremendous potential to be affected compared to other aquatic organisms. It was observed that exposure to filtered TiO_2_ NPs and C_60_ caused mortality, in which the latter caused toxicity at much lower concentrations. The LC_50_ for TiO_2_ NPs was 5.5 ppm while that of C_60_ reached a much lower dose of 460 ppb. This difference was related to the NPs’ size since the C_60_ (0.72 nm) was significantly lower than TiO_2_ NPs (10–20 nm). Also, the adopted synthesis method of NPs has been found to affect their toxicity. Unfiltered sonicated NPs caused little mortality; at 25–30 times higher concentrations of TiO_2_ NPs, there was not much difference compared to the control. Furthermore, at a concentration of 500 ppm C_60_, there was only 18% mortality. These effects may result from the presence of larger NPs and clusters [134]. 

Algae are also essential species of the aquatic environment, as they play an indispensable role as food sources and photosynthesis. The effects of Fe_3_O_4_ NPs were evaluated in the microalgae *Chlorella vulgaris*. The results showed changes in chlorophyll content, reduced CO_2_ absorption, and photosynthetic rate from 50 mg/L. Oxidative stress was also noticed due to changes in stress markers’ levels. The increased levels of malondialdehyde, related to lipid peroxidation; and the reduction in glutathione levels, an antioxidant molecule that eliminates reactive oxygen species (ROS), demonstrated this finding [135]. 

In another study, the toxicity of Ag, Cu, Co, Ni, Al, and TiO_2_ NPs were tested against zebrafish (*Danio rerio*), adult specimens of *Daphnia pulex*, neonates of *Ceriodaphnia dubia*, and algae (*Pseudokirchneriella subcapitata*) to better understand the effects at various trophic levels. Besides, soluble salts were also tested to compare the difference between the effects of NPs and salts. It was found that both Ag- and CuNPs were toxic to all tested organisms, with the calculated LC_50_ ranging from 0.04 mg/L (*D. pulex*) to 7.2 mg/L (*D. rerio* fry) for AgNPs and 0.06 mg/L (*D. pulex*) at 0.94 mg/L (adults *D. rerio*) for CuNPs. *Daphnia pulex* was also susceptible to NiNPs (LC_50_, 3.89 mg/L). All tested NPs caused toxicity in *C. dubia* and *P. kirchneriella* at 48 and 96 h of exposure, respectively. TiO_2_NPs showed no toxicity in any of the tests. Based on these results, invertebrates were markedly more susceptible to NMs’ toxicity than any zebrafish stage. In general, metal salt solutions were more toxic than NPs forms [136].

### 5.2. Terrestrial Environments

In terrestrial ecosystems, NMs can directly or indirectly affect plants, microbial communities, and soil organisms. In plants, they can affect plant seeds’ germination and the development of plant roots and sprouts. NMs’ interaction with plants induces several physiological and morphological modifications including impaired seed germination, plant growth inhibition, reduced biomass production, and imbalanced nutrients as well as an alteration in photosynthesis and transpiration rates [137]. NMs present in the environment upon interaction with plants can be internalized in plant tissues either through roots or above-ground parts [138]. In addition to terrestrial plants, NMs can affect the growth of soil microorganisms, and invertebrate animals such as snails, earthworms, or larvae insects. Besides, contaminating groundwater and being absorbed by crops, and possibly entering the food chain with potential damage to animals and humans’ health [10,86,139].

AgNPs could inhibit *Phaseolus radiatus* and *Sorghum bicolor* growth in an agar medium, with a concentration-dependent effect. The growth rate was not affected when tested on the soil. The accumulation of Ag in the root cells was also observed [140]. The effects of CuNPs were also evaluated in wheat (*Triticum aestivum* L.). The plant’s growth rate decreased markedly up to 60%, and the formation of lateral roots was stimulated. Oxidative stress associated with the presence of CuNPs has been reported [141]. Another study evaluated the effect of fullerene C_70_ and MWCNTs overall rice growth stages. Black aggregates were found frequently in seeds and roots and less regularly in stems and leaves, indicating that the NPs’ absorption sequence was from the plant’s seeds and roots to the stems and leaves. The appearance of black aggregates mainly within and near the stem’s vascular system suggests that the transport of C_70_ occurred simultaneously with the absorption of water and nutrients in the xylem. However, the capture of MWCNTs was of little relevance, with few black aggregates appearing in the vascular system and almost none in plant tissues [142]. 

When exposed to CuONPs for 15 days, cabbage (*B. oleracea var. capitata* L.) and lettuce (*L. sativa* L. *cv. Batavia*) showed a decrease in plant weight and photosynthesis level. Additionally, CuONPs affect the stomata and were translocated to roots [143]. Three different NPs (ZnO, CuO, or CeO_2_) and their equivalent ions (Zn^2+^, Cu^2+^, or Ce^4+^) were tested in sweet potato (*Ipomoea batatas*) development. The CeO_2_NPs caused a small positive effect at a higher concentration (1000 mg·kg of dry weight^−1^) on tuber biomass, while the ZnO and CuONPs caused negative effects on tuber biomass at the same concentration. Additionally, the accumulation in both the peels and flesh was observed for all metals with no difference between the NMs and ionic treatments. This suggests that the dissolution of the NPs took place before the accumulation process [144]. The growth of the rice plant (*Oryza sativa* L.) was strongly affected by CuONPs, reducing the water content in the shoots and roots; at a higher concentration (1000 mg·kg of dry weight^−1^) no plant survived to all stages, and at lower concentrations (500 mg·kg of dry weight^−1^), the fresh weight of grains was reduced to only 6.51% of the control values. The accumulation in the tissue plants was also seen [145]. Furthermore, AgNPs caused more toxicity to lettuce by root exposure than foliar, and ultrastructural injuries, oxidative damage, accumulation in tissues, and biomass reduction were observed [146]. 

In wheat growth under hydroponic conditions, Fe_3_O_4_ NPs were detected in root cells, but no damage to the plant was observed. The NPs did not alter the chlorophyll content, plant growth, or germination rate and no oxidative damage or lipid peroxidation was noticed [147]. When comparing the effects of AgNPs to Ag^+^ on *Arabidopsis thaliana*, it was reported that AgNPs were more toxic. The AgNPs accumulated in the leaf cells inhibited root elongation, decreased chlorophyll content, and altered the transcription of antioxidant and aquaporin genes, affecting water homeostasis and antioxidant systems [148]. Similar results were observed in potatoes (*Solanum tuberosum* L.) treated with AgNPs and Ag^+^. Despite the Ag content in ion-treated plants being higher than the ones treated with AgNPs, the last caused significantly higher oxidative stress and a decrease in the glutathione and ascorbate contents [149].

In contrast, the ionic form of Ag was more toxic than any AgNPs with different surface coatings (citrate, PVP, and cetyltrimethylammonium bromide (CTAB)) tested on *Allium cepa* roots. These AgNPs exhibited toxicity only at higher concentrations. AgNP-CTAB led to bigger toxicity, causing root growth inhibition and oxidative stress. The larger particle AgNP-citrate demonstrated a tendency to aggregate, thus the effects were less pronounced [150]. Moreover, both *Aloe vera* biosynthesized AgNPs and AgNO_3_ reduced the growth of *Brassica* seedlings. This occurred due to the accumulation of Ag, which caused photosynthesis inhibition. Additionally, both led to oxidative stress, associated with increased DNA degradation and cell death. However, AgNPs were less toxic than AgNO_3_ due to less accumulation and better activities of the antioxidant enzymes [151]. In a similar approach, the ZnONPs, bulk ZnO, and ZnCl_2_ were tested against the symbiotic Alfalfa (*Medicago sativa* L.)—*Sinorhizobium meliloti* association. ZnONPs reduced the dry root biomass by 80% in all tested plants and ZnCl_2_ was reduced by 70–87%. Conversely, bulk ZnO increased the shoot and root biomass, while the ZnONPs increased the root length at 250 mg/kg soil, but at higher concentrations (500 and 750 mg/kg), no significant changes were observed. ZnCl_2_ reduced the total leaf protein in a dose-dependent and catalase activity in the stems and leaves at higher concentrations. Thus, ZnCl_2_ was more toxic than the ZnONPs and bulk ZnO could enhance the plant’s growth [152].

Three different carbonaceous nanomaterials, MWCNTs, graphene nanoplatelets (GNPs), or carbon black (CB) NPs were tested in the soybean plant. The soybean was grown for 39 days in soil amended with these NMs. The treated plants flowered earlier and produced up to 60–372% more flowers. In general, there were no considerable effects on the stem length, but the MWCNT-treated plants and leaves were shorter. This effect may have been in response to a stressful environment, which accelerated plant flowering to maximize the chance of flowering, at the cost of the final plant height and leaf area. Additionally, N_2_ fixation potential was reduced in all treatments, mainly for CB and MWCNT. In addition, CB and GNPs accumulated inside the root nodules [153].

Positive effects were also observed. Biosynthesized AgNPs significantly increased the root length, shoot length, fresh weight, and dry weight of rice seedlings compared to the control [154]. After foliar application in the fenugreek plant (*Trigonella foenum-graecum*), AgNPs promoted the growth, chlorophyll, and carotenoid content. In addition, it enhanced the number of pods and seeds and increased the antioxidant activity of the yielded seeds [155]. Other reports showed similar effects of AgNPs in the fenugreek plant, showing a considerable effect on its growth parameters such as leaf number, root length, shoot length, and wet weight [156]. 

Commercially available uncoated ZnONPs (Z-COTE) and coated (Z-COTE HP1) did not affect bean (*Phaseolus vulgaris*) germination and pod production. While Z-COTE^®^ NPs did not lead to phenotypic changes, the Z-COTE HP1^®^ NPs affected the root (~44%) and leaf length (~13%). The plant’s exposure to 125 mg/kg of Z-COTE^®^ NPs increased the Zn content by 203%, 139%, and 76% in the nodules, stems, and leaves, respectively, while at the same concentration, Z-COTE HP1^®^ increased Zn by 89%, 97%, and 103% in the roots, stems, and leaves, respectively. Moreover, the ZnCl_2_ treatment reduced the chlorophyll content by 34–46% and the leaf length by 16% at 125 mg/kg, while the bulk ZnO reduced the root length by 53% at 62.5 mg/kg [157]. 

PVP-coated CeO_2_NPs enhanced the plant’s fresh weight at all concentrations, while uncoated CeO_2_NPs only increased at higher concentrations. Both NPs increased the water use efficiency at low doses but decreased at higher ones. There were no significant differences in the total chlorophyll content, but when chlorophyll *a* and *b* were compared separately, changes could be noted for the PVP-CeO_2_NPs. For these NPs, the chlorophyll *a* content increased but not the chlorophyll *b*. Both CeO_2_NPs stimulated the photosynthesis rates, which varied depending on the time exposure or dose. This suggests that the mechanism of this enhancement could vary. Additionally, uncoated-CeO_2_NPs led to a higher Ce content due to their opposite surface charges between PVP-CeO_2_ (negatively surface) NPs and the uncoated-CeO_2_NPs (positively charged) [158]. *Thuja occidentalis* extract–mediated green AgNPs improved the chlorophyll content, leaf number and area index, and nitrate reductase activity in *Phaseolus vulgaris*. However, higher doses (50 and 100 mg⋅kg) increased the proline levels, indicating higher oxidative stress [159]. 

Comparing the C3 plants (wheat and rice), C4 plants (*Amaranthus tricolor* L.), and maize (*Zea mays Linn*), the last were found to be more resistant to NM exposure. Important changes in the fresh weight were observed for rice with the fresh weights being reduced by 43%, 47%, 28%, 46%, and 52% for the CuO, Ag, TiO_2_, MWCNT, and few-layer graphene (FLG) NMs, respectively. In the case of wheat, the fresh weight was not significantly affected. In the case of the C4 plants, the fresh weight of maize was reduced across all NM exposure by 22%, 28%, 20%, 27%, and 15% for the CuO, Ag, TiO_2_, MWCNT, and FLG NMs, respectively. In the case of amaranth, the fresh weight was reduced by 41%, 19%, 23%, and 18% for the CuO, Ag, TiO_2_, and FLG NMs, respectively. In the MWCNTs, the fresh plant weight increased by 77%. A similar trend was also observed for root elongation, with the overall effect on C3 plants being the highest. In the case of the C4 plants, no significant effects on the roots were observed for amaranth, whereas Ag, TiO_2_, and FLG reduced the root elongation of maize by 35%, 26%, and 33%, respectively. The C3 plants reduced root elongation by 29%, 21%, and 29% for Ag, TiO_2_, and MWCNTs in wheat and 29%, 37%, and 29% for CuO, respectively, Ag- and TiO_2_NPs in rice. NM uptake and metal variation were greater in C3 species than in the C4 plants. For example, Ag concentrations in wheat and rice were at least 1.3-fold higher than that manifested in maize and amaranth. Copper levels in rice were 1.4- and 1.6-fold higher than in maize and amaranth, respectively. The copper concentrations in wheat were 1.3- and 1.5-fold greater than in maize and amaranth, respectively. The titanium content in rice was 1.6- and 3.5-fold higher than in maize and amaranth, respectively. In rice, CuONPs affected the chloroplast thylakoid structure and accumulated in the vascular sheath cells. In contrast, there were no considerable changes in the chloroplasts, and accumulation was found in the maize leaves treated with CuONPs [160]. 

### 5.3. Human and Animal Health

**Figure 5:**
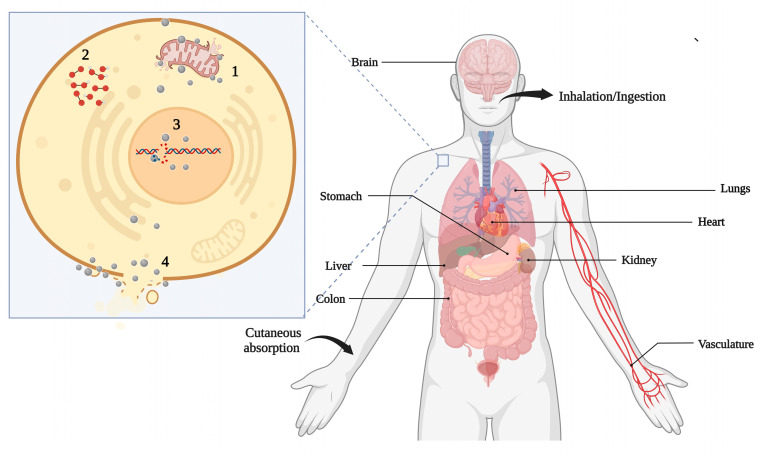
Major possible consequences of the toxicity of nanomaterials to human health. The main routes of exposure are ingestion, inhalation, and absorption through the skin, which can cause damage to different organs such as the lungs, kidneys, heart, stomach, and liver. At the cellular level, nanomaterials are believed to affect cells through mitochondrial damage (1), ROS production (2), DNA and protein damage (3) as well as cell membrane disruption (4).

The effects of NM exposure on human and animal health have also been investigated. The mechanisms related to the toxicity of NMs vary depending on the studied cells and NM. In general, cytotoxicity is related to increased reactive oxygen species levels, decreased membrane potential, and antioxidant levels. DNA and proteins can also be affected directly or by the produced reactive species (Figure 5). DNA damage can cause the cell cycle to stop in the G2/M phase. Damage to the cell membrane can leak the cytoplasmic content and eventual necrosis, while rupture of the lysosomal membranes activates lysosome-mediated apoptosis [93,161]. 

Human and animal exposure is usually due to food, air, medical applications, and water. Exposure routes influence potential risks including inhalation, cutaneous absorption, ingestion, and injection (Figure 5). Unfortunately, there is little information about the levels of exposure to workers and the public [162]. Ingestion can usually occur through the consumption of contaminated food and water. The main target of the inhalation route is the respiratory tract. The lung is one of the main targets including the pulmonary epithelial cells and cells of the immune system and fibroblasts. It is essential in response to NM exposure and related inflammatory conditions, fibrosis, and genotoxicity induced by these NMs [10]. NPs in cosmetics are an important route of exposure for the skin. They often reach systemic circulation, in which several organs can be affected such as the liver, kidney, heart, and consequently become target organs [113]. 

The effects of starch-coated AgNPs were evaluated in normal human lung fibroblasts (IMR-90) and human glioblastoma cells (U251). Exposure to AgNPs caused damage to the mitochondria and increased reactive oxygen species (ROS). DNA damage was also observed. However, the tumor cell appeared to be more affected. This damage were possibly responsible for stopping the cell cycle in the G2/M phase. AgNPs were found deposited inside the mitochondria and nucleus, which can be directly related to DNA damage and mitochondrial toxicity [163]. The cytotoxicity of AgNPs synthesized using the fungi *Trichoderma viride* was evaluated with an environmentally friendly proposal. They were able to significantly inhibit the proliferation of the MCF-7 cancer cell lines in a time- and concentration-dependent manner. DNA fragmentation was also observed, and at a concentration of 100 μg/mL, the AgNPs exhibited significant cytotoxic effects alongside apoptotic characteristics. These effects against cancer cells demonstrate their chemotherapeutic potential [164]. Silver NPs biosynthesized with European black elderberry extracts (*Sambucus nigra*) showed anti-inflammatory potential in models of acute inflammation and lesions caused by psoriasis in humans. Morphological changes in the concentration-dependent trend of HaCaT keratinocytes were observed. The cells became rounded, losing their characteristic epithelial cell shape and intense cytoplasmic vacuolization. These NPs accumulated in the keratinocytes, and cell viability was reduced by 50% at 79.4 µg/mL [33].

It is important to highlight that the results reported in the literature vary widely depending on the used NMs. CNTs did not lead to acute toxicity in the cell viability assays in rat NR8383 lung cells and human A549. However, increased intracellular reactive oxygen species (ROS) concentration and decreased mitochondrial membrane potential were observed [165]. Halloysite nanotubes (HNTs) and MWCNTs were tested in human umbilical vein endothelial cells (HUVECs) in vitro and in the blood vessels of mice in vivo. After exposure to these NMs, HUVECs reduced the cellular viability while enhancing the apoptotic proteins and adhesion molecules, mainly in MWCNTs. HNTs generated significant cytotoxicity only at the concentration of 128 µg/mL, while MWCNTs were more toxic, showing cytotoxicity at 32, 64, and 128 µg/mL. Moreover, the adhesion VCAM-1 protein levels increased in both treatments with higher effects for MWCNTs. Additionally, the NMs were internalized by these cells. When the NMs were injected intravenously (50 μg/mouse, once a day for five days), the percentage of neutrophils, monocytes, and basophils increased. Furthermore, they led to autophagic dysfunction, activation of apoptotic proteins, and decoupling of endothelial oxide nitric synthase (eNOS) involved in fundamental processes such as cellular proliferation and platelet aggregation [166].

AgNPs subjected to the action of simulated saliva, gastric, and intestinal fluids showed changes in size and morphology depending on the fluid’s pH and composition. The main effect observed was aggregation, thus increasing the size of the NPs. However, low toxicity was observed for the 3D models of these tissues. Furthermore, inflammatory cytokine IL-1a decreased in epithelium models of gingival and oral tissue after repeated application of AgNPs, thus manifesting anti-inflammatory properties [167]. Synthetic human body fluids were also used to investigate the chemical transformations of AgNPs. In gastric acid fluid (pH 1.5), accelerated dissolution occurred, but was incomplete for most particles due to limited contact time (10–240 min in the stomach). The Ag^+^ can interact with Cl^−^ to form AgCl, and in the gastric acid fluid, it enters the bloodstream through systemic circulation by active transport routes for Na^+^ and enter the bloodstream. It is expected to bind to proteins like albumin in thiol complexes and be distributed to various tissues and organs. Additionally, it was observed that Ag-thiol complexes under UV light are photo-reduced and form new AgNPs that can react with sulfur (S) and selenium (Se). Fascinating findings were Se tarnishing of Ag surfaces and the selenide’s capacity to quickly replace sulfide in Ag_2_SNPs and Ag_2_S films through exchange reaction, leading to argyria through deposits as Ag/S/Se are located in the near-skin region. This deposition occurred preferentially in the light-affected areas [168].

Recent studies have shown that NMs can also affect gene transcription and have epigenetic effects [169,170]. One of the first studies to suggest that NMs could affect the cell’s epigenome evaluated the effects of SiO_2_ of 15 nm in the human epidermal keratinocyte cell line HaCaT. A decrease of more than 20% in global DNA methylation was observed. In addition, the levels of methyltransferases (DNMTs) and methyl-CpG binding protein 2 also decreased [171]. AgNPs were able to cause a great inhibition of RNA polymerase activity and of transcription as a whole through the Ag binding to RNA polymerase. Mouse erythroleukemia cells treated with a concentration of 1 μg/mL of different AgNPs (10, 25, 40, or 110 nm spherical and 45 nm plate-like) showed a large reduction in α-globin and β-globin mRNA levels. However, these cells did not show any reduced cell viability. The most pronounced effects were seen in AgNPs (spherical) and those with smaller sizes (10 and 25 nm). This may be related to the greater surface area available for interaction and dissolution as well as greater uptake by the cells. These effects were not dose-dependent (1, 4, and 8 μg/mL) and the Fe levels, which are critical for erythropoiesis and hemoglobin production, were not significantly affected. Overall, the mRNA levels in these cells were also reduced and the acellular runoff transcriptional assay in the presence of the Ag nanomaterial demonstrated a reduction in RNA synthesis, thereby demonstrating that the effects could be directly on RNA polymerase. To differentiate whether the effects were due to AgNPs or Ag ions, MEL cells treated with Ag ions (in AgNO_3_) were vulnerable at even low concentrations (0.08 μg/mL), with a significant reduction in cell viability. Although Ag ions could impair the cell viability at concentrations of 0.08 and 0.80 μg/mL, globin gene transcription was not significantly affected compared to the Ag nanomaterial at 8 μg/mL. Additionally, it was demonstrated that over 82.1% of Ag existed in the form of NPs, with less than 17.9% as Ag ions inside MEL cells. Furthermore, the AgNPs could potentially bind to RNA polymerase but not to DNA in the in vitro transcriptional system, leading to impaired Sp6 RNA polymerase activity and reduced RNA synthesis. In vivo studies with mice also showed a large reduction associated with anemia in the embryos. The above findings showed that the Ag nanomaterial can change transcription levels through RNA polymerase inhibition [172]. Similar effects were observed for CdSe quantum dots (QDs) that could accumulate in the embryonic hematopoietic organ fetal liver, inducing a significant reduction in hemoglobin mRNA transcription in these liver cells. This decline was a consequence of the inhibition of RNA polymerase function [173]. Another study reported that AgNPs reduced the hemoglobin levels in erythroid cells. This effect was associated with a considerable reduction in the global methylation level for histone H3, mainly in Lys 4 and Lys 79, at sublethal concentrations and without oxidative stress. The changes in methylation were attributed to the reduced histone methyltransferase levels and the direct binding between AgNPs and the histone, as predicted by the all-atom molecular dynamic simulations [174]. In another approach, (5, 60, and 250 nm) AuNPs were administered intra-tracheal at low (0.25 mg/kg) and high (2.5 mg/kg) doses to assess the effects on the methylation pattern of cells. No effects on global DNA methylation were observed, but 60 nm AuNPs induced CpG hypermethylation (*Atm, Cdk*, and *Gsr*) and hypomethylation (*Gpx*) in different genes. Large AuNPs (250 nm) also changed *Trp53* methylation, while 5 nm AuNPs did not significantly affect CpG methylation compared to the controls [175]. Spherical AgNPs and an average particle size of 8 nm were administered to pregnant mice via intravenous infusion at 1.0 mg/kg doses at 6.5 days *post coitum* (dpc) and euthanized after 13.5–17.5 dpc. AgNP treatment enhanced the meiotic progression of female germ cells in the fetal mouse ovaries. The RNA levels of some development-related genes (*Cx37*, *Zp1*, *Zp2*, *Zp3*, and *Fliga*) of oocytes were significantly reduced after exposure. The treatment also disrupted imprinted gene expression (*Ascl2*, *Snrpn*, *Kcnq1ot1*, *Peg3*, *Zac1*, *H19*, *Igf2r*, and *Igf2*) in the embryos and placentas [176].

In contrast, two types of carbon dots (CDs, one positively charged and the other negatively charged) did not cause changes in the DNA methylation pattern in human embryonic lung fibroblasts (HEL 12469), even when exposed to concentrations of 50 and 100 µg/mL, respectively, for a long duration of 24 h. However, miRNA and mRNA expressions were affected by other mechanisms associated with immune response, tumorigenesis, and cell cycle regulation after exposure to positively charged CDs. For the negatively charged CDs, it was associated with pathways related to cell proliferation, apoptosis, oxidative stress, gene expression, and cycle regulation [177]. The mechanisms of epigenetic alterations after NM exposure are not clear. However, we can presume that these alterations occur by altered functioning of chromatin-modifying proteins, oxidative stress, and inflammation, and the reduction in antioxidant glutathione levels that lead to the demethylation of DNA and histone alterations [170]. Moreover, various pathways could be involved in these changes.

### 5.4. Impacts on the Food Chain

The risks also need to be evaluated across the entire food chain, considering the trophic transfer potential. A recent study evaluated the impacts and distribution of AuNPs at different trophic levels in an aquatic food chain. Initially, the algae of the species *Pseudokirchinella subcapitata* were exposed to AuNPs of different shapes and sizes. For a concentration of 2.9 × 10^11^ particles·mL^−1^, no toxicity was observed in the algae subjected to these exposures. However, it was observed that 10 nm spherical NPs were present in 68% of the cells in the algal population. The rod-shaped 10 × 45 nm NPs were present in just 34% of the cells. In daphnids fed with AuNP-exposed algae, the Au accumulation was 0.73% (for spherical) and 1.71% (for rod-shaped) of the total Au in algae [178].

At the next trophic level, zebrafish were fed with 10 contaminated daphnids each day for 21 days. A small percentage of AuNPs accumulated in daphnids transferred to zebrafish varied from 0.03% (for spherical) to 0.48% (for rod-shaped) with the brain and liver as the target organs. Furthermore, AuNP dissolution and agglomeration in the gut of the daphnids and biodistribution in fish tissues were found to be size- and shape-dependent [178]. Similarly, positively charged 10-nm diameter AuNPs functionalized with polyethylene oxide demonstrated potential for trophic transfer from periphytic biofilms to the crustacean *Gammarus fossarum*. Biofilms exposed for 48 h to AuNPs at two concentrations, 4.6 and 46 mg/L, were used to feed the crustacean for 7 days, with daily biofilm renewal. Both approaches generated oxidative stress in cells, mainly affecting mitochondrial respiration. Alterations in digestive enzyme activity were also reported. In the cilia of apical intestinal epithelial cells, muscle fibers, and mitochondria, the latter clearly showed a disruption of crests and internal and external membranes. Moreover, a significant difference in expression in three genes occurred: cat (catalase) and sodMn (superoxide dismutase) associated with oxidative stress; and 16S associated with mitochondrial metabolism. On the other hand, no differences in the number of metallothioneins were reported. Nevertheless, the amylase and trypsin activities were 1.45-times higher when gammarids were exposed to the highest AuNPs concentration in biofilms (46.5 mg/L) [179].

In another study, C^13^-fullerenol NPs were tested in marine ecosystems. *Scenedesmus obliquus* (phytoplankton) was exposed to C^13^-fullerenol (1 mg/L) for 36 h under continuous gentle aeration. These NPs accumulated in *Scenedesmus obliquus* through water exposure, where it was possible to observe black dots of 18 to 78 nm in algal cells. In *Daphnia magna* fed with *S. obliquus*, no mortality was observed. When ingested by zebrafish, fullerenol accumulated more in the intestine, followed by the liver, muscle, gills, and brain. After the 28 days of uptake and a 15 day depuration period, fullerenol NPs were mainly distributed in the intestine and liver. The gastrointestinal tract is the initial target tissue in dietary exposure, and it is expected that fullerenols can pass through intestinal membranes and enter the circulatory system, where they are subsequently distributed to other tissues. The biomagnification factor (BMF) value of fullerenes from *S. obliquus* to *D. magna* was 3.20, while the BMF value from *D. magna* to *D. rerio* was 0.54. This value is dependent on the gut passage time, assimilation, elimination ratio, and environmental conditions. Thus, the biomagnification of fullerenes was higher from the first to the second trophic level, but not to the next trophic level [180]. For the CdSe/ZnS QDs, the results indicated that QDs could transfer from zooplankton to *D. rerio* by dietary exposure. However, no considerable biomagnification was reported and the BMF for both adult and juvenile zebrafish was less than one (0.04 and 0.004, respectively). In addition, no mortality was observed [181].

Moreover, the effects of carbon nanofibers (CNFs) across trophic levels were evaluated in a food chain: *Eisenia fetida/D. rerio/Oreochromis niloticus*. *E. fetida* individuals were exposed to CNFs and offered to *D. rerio*, later feeding *O. niloticus*. The accumulation and cytotoxic effects occurred in *O. niloticus* exposed to CNFs via the food chain, and it was associated with an elevated number of erythrocyte nuclear abnormalities such as constricted erythrocyte nuclei, vacuole, blebbed, kidney-shaped, and micronucleated erythrocytes. These results suggest the occurrence of defects caused by CNFs in cell division processes. In contrast, the presence of blebbed, kidney-shaped, and notched nuclei can be inferred as precursor changes to micronuclei. Oxidative stress induction by CNFs is another possibility that could explain the erythrocyte nuclear abnormalities observed in this study [182].

In another approach, the toxicity and trophic transfer potential of TiO_2_NPs from marine algae *Dunaliella salina* to marine crustacean *Artemia salina* were evaluated by waterborne (exposure of TiO_2_ NPs to *Artemia*) and dietary exposure (NPs-accumulated algal cells are used to feed the *Artemia*). The crustacean was more susceptible to TiO_2_NPs (48 h LC_50_ of 4.21 mg/L) than marine algae, *D. salina* (48 h LC_50_ of 11.35 mg/L). A decrease in chlorophyll content dependent on the exposure concentration was also observed, which correlated well with the cell viability loss results. The toxicity, uptake, and accumulation were higher with waterborne exposure compared to dietary exposure. No trophic transfer from algae to *Artemia* occurred through dietary exposure. Waterborne exposure seemed to cause higher ROS and antioxidant enzyme production, accumulating in the midgut region, and caused the deformation of cells in the epithelial lining of the hindgut region [183].

The potential of PEG-coated AuNPs in the trophic transfer was evaluated using an experimental benthic food chain that included two trophic levels: natural river biofilm and grazer fish *Hypostomus plecostomus*. For 21 days, the fish were exposed to the biofilms earlier contaminated with 0.48 or 4.8 mg/L AuNPs. No fish mortality was noted during this experiment. The Au content could be transferred from the biofilms to fish and the bioaccumulation was organ and exposure level-dependent. All organs except the heart showed a significant Au accumulation. The digestive tract had the highest accumulation, 60- and 220-fold more than the control for 0.48 and 4.8 mg/L AuNP exposure. The kidney was second with 49- and 154-fold increases in 0.48 and 4.8 mg/L compared to the control. The brain significantly increased the gold concentration at the highest exposure level, 5-fold more than the control, whereas 0.48 mg/L was unchanged. Even in lower concentrations, histological alterations were detected in the exposed liver, spleen, and muscle of the fish. The liver exhibited a severe increase in the lipid droplets in the size and frequency with vacuolization of the hepatocyte cytoplasm. In the spleen, the control fish showed a homogeneous distribution of cell types, whereas melano-macrophage centers were observed in the exposed fish. Skeletal muscle tissue appeared disordered for the exposed fish compared to the control, with disorganized myofibrils and broken fibers leading to spaces between the fiber bundles [184].

In another study, the phytoplankton *Chlamydomonas reinhardtii* was exposed to AgNPs (citrate-coated and fluorogen-coated) with a concentration of 1000 μg/L. The phytoplankton internalized both, but the surface coating changed the entrance pathways. Fluorogen-coated AgNPs were internalized through endocytosis and citrate-coated AgNPs through the apical zone of the cell near the flagella. In the cells, AgNPs suffer dissolution, releasing Ag ions in the cytoplasm. The trophically transferred AgNPs to *D. magna* were concentrated in the gut regions due to the direct ingestion of food particles. After ingestion, about 95% of the trophically transferred Ag^+^ was eliminated. Retention of fluorogen-coated AgNPs by daphnids was more intense than that of the citrate-AgNPs due to their lower dissolution of Ag^+^ [185]. The ecotoxicological effects of silver nanowires (AgNWs) were evaluated on freshwater organisms and their transfer through the food webs. AgNWs of 10 and 20 m long were tested on the alga *Chlamydomonas reinhardtii*, *Daphnia magna*, and zebrafish. It was observed that longer AgNWs were more toxic than shorter ones to both the algae and water fleas, but shorter AgNWs accumulated more than longer ones in the fish’s body (Chae and An, 2016).

NM toxicity through food chains in terrestrial ecosystems was studied in a soil earthworm (*Eisenia andrei*)—Collembola (*Lobella sokamensis*) food chain. The survival earthworms were assessed after the 7 days of exposition to AgNP contaminated soil (50, 200, and 500 µg AgNP/g soil dry weight). After this time, they were killed and added to the receptacles with *L. sokamensis*. At the lowest concentration, low bioaccumulation and negligible effects were reported. Nonetheless, the highest concentration led to juvenile mortality and enhanced the trophic transfer to Collembola, which impaired the locomotion and survival rates in a dose-dependent manner [186].

In another food chain model, *Escherichia coli* (*E. coli*) and *Caenorhabditis elegans* (*C. elegans*) were used to assess the PVP-coated AgNP toxicity. The AgNPs accumulated in *E. coli* and transferred to the *C. elegans* through the food chain. The AgNPs were in the gut lumen, subcutaneous tissue, and gonad, and induced extreme toxicity including effects on reproductive integrity and life span, and germ cell death. Relative to larger NPs (75 nm), small ones (25 nm) accumulated more easily and were more toxic to the higher trophic level. This finding reinforces that smaller NMs have more toxicity (Luo et al., 2016). The trophic transfer potential of AuNPs was evaluated using the plant *Nicotiana tabacum* L. *Cv Xanthi* and the caterpillars (*Manduca sexta*). The BMF of the primary producer to a direct consumer was 6.2, 11.6, and 9.6 for the 5, 10, and 15 nm AuNP treatments, respectively [187]. The same organism (*Manduca sexta*) was fed with tomato leaves contaminated with 12 nm tannate-coated AuNPs. A low bioaccumulation factor (BAF) of 0.16 was observed and no evidence that the caterpillars were affected by the ingestion of AuNP contaminated plant tissue, indicating that the bioaccumulation efficiency via this pathway is not considerable for this study model [188]. The differences in the obtained results may originate from the different routes of exposure of the caterpillar. In the first one, there were NPs in the plant tissues, while in the second, the NPs were on the surface of the leaves.

Another point that needs to be considered when evaluating toxicity is the effect of NMs in the presence of other contaminants. NMs could serve as carriers for other contaminants, enhancing the absorption by organisms, reactivity, bioavailability, or even present synergistic effects. However, the interaction of NMs with contaminants can also lead to positive effects. NMs can interact and adsorb contaminants, decreasing their availability, leading or not to aggregation and sedimentation, promoting the degradation of contaminants, and competing with contaminants for interaction sites [189]. MWCNTs were not toxic to freshwater fish Nile tilapia (*Oreochromis niloticus*), but they potentiate the toxicity of the pesticide carbofuran more than 5-fold [190]. Five types of CNTs manifested varied results such as increasing or reducing toxicity against *D. magna* in the presence of the herbicide phenanthrene, depending on the condition evaluated [191]. In addition, CNTs with co-contaminant Pb caused severe histopathologic alterations in the gills of Nile tilapia such as hyperplasia and the displacement of epithelial cells [192]. MWCNTs also promoted positive effects on organisms. MWCNTs reduced the toxicity of antimicrobial triclocarban against three cell lines: rainbow trout liver cells (RTL-W1), human adrenocortical carcinoma cells (T47Dluc), and human adenocarcinoma cells (H295R). The lethality of this biocide was also reduced against *D. magna* when incubated with MWCNTs [193]. Fullerene C60 reduced the toxicity of pentachlorophenol by 25% in tests with *D. magna*. However, it increased more than ten times the toxicity of phenanthrene. No difference was observed for atrazine or methyl parathion [194].

**Figure 6:**
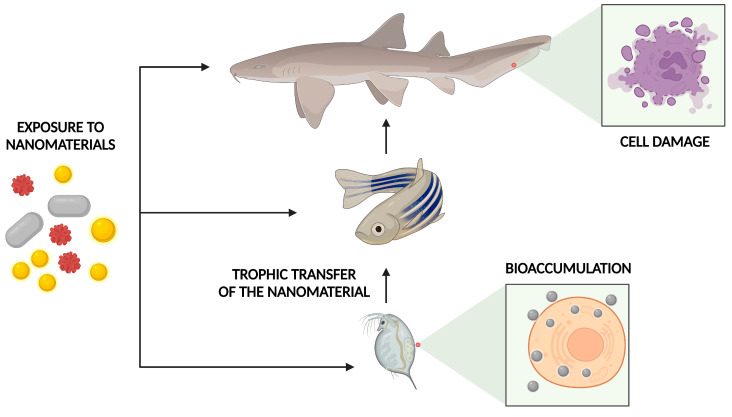
NMs can cause harm associated with acute and chronic exposure that can affect the entire food chain. NMs can directly affect each member of a food chain and accumulate in their tissues. Furthermore, this accumulation can be transferred through the trophic levels, causing even greater damage, mainly to the top predator of the chain.

No developed effects of TiO_2_NPs were observed at 2 mg/L in embryonic abalone (*Haliotis diversicolor supertexta*) but at the same concentration, the TiO_2_ increased the toxicity of tributyltin up to 20-fold compared with tributyltin alone, causing malformation. This enhancement was associated with tributyltin adsorption onto TiO_2_NP aggregates and the internalization of them by abalone embryos [195]. TiO_2_NPs also increased the toxicity of phenanthrene and cadmium (Cd^2+^) to *A. salina* at a low concentration of 5 mg/L TiO_2_NPs. However, at higher concentrations of 400 mg/L, the toxicity decreased by 24.5 and 57.1%, respectively. This toxicity can be associated with the pollutants’ adsorption on the surface of the TiO_2_NPs, TiO_2_-facilitated bioaccumulation of pollutants, and the aggregation and sedimentation of TiO_2_NPs [196]. In addition, TiO_2_NPs promoted the metabolism of pentachlorophenol to tetrachlorohydroquinone in zebrafish larvae, and the co-exposure led to higher ROS generation, eventually leading to lipid peroxidation and DNA damage [197]. In addition, TiO_2_NPs could facilitate arsenic (As) sorption on microalgae *Nannochloropsis maritima* within an exposure period of 24 h. This effect caused a higher As trophic transfer from the algae to *A. salina nauplii*. However, the retention of As in *A. salina* fed As-TiO_2_NP contaminated algae was lower than one fed As-contaminated algae after 48 h of depuration. This could be due to the reduced bioavailability of As in the presence of TiO_2_NPs [198]. In another study, a higher affinity of Cd(II) toward TiO_2_ was observed. The presence of TiO_2_NPs improved the accumulation of Cd in carp (*Cyprinus carpio*). After 25 days of treatment, Cd concentration in carp significantly increased by 146% with a positive correlation between the Cd and TiO_2_ concentrations [199]. In summary, nanomaterials can affect biological systems through either acute or chronic exposure. As presented above, this exposure can cause to damage to genetic material and organelles such as mitochondria and proteins, leading to cell death. In addition, some studies have shown that accumulation and its effects can all be transferred along the food chain (Figure 6).

## 6. Challenges

Assessing the toxicity of NMs still presents many challenges, although some advancements in research and regulations have been made. As previously mentioned, several parameters influence the characteristics of NMs, and consequently their toxicity. Furthermore, there exists a discrepancy in the limits when comparing between study results and the creation of quality standards [200]. To better understand the actual risks, the ideal would require evaluating the long-term effects and mimicking the environments NM could contaminate to bring them closer to the natural environment. However, many details of NM interactions with biotic and abiotic factors are unknown [201]. In addition, more studies are necessary to determine the long-term stability of NMs and the associated toxicity.

Since the toxicity of NMs varies according to the different physicochemical characteristics alongside the existence of different results regarding their toxicity, understanding these dynamics and establishing patterns remains a great challenge. Therefore, it is vital to consider the specific properties of each material aside from the exposure routes and the corresponding conditions. Many steps can be taken into consideration to better manage the risks of nanotechnology. The first step in assessing the potential risks of a nanomaterial is to perform in-depth and breadth characterizations. Studying its physicochemical characteristics will help to estimate the possible effects on a biological system and elucidate more precisely which ones can be modified to reduce toxicity.

To understand the effects on biological systems, it is important to have a well-defined nanomaterial concentration and characterization. Fluorescent, isotopic, or radio-labeled markers could be used to determine the dosage, meanwhile, the labeling process can change the biodistribution and physicochemical properties as well as contribute to the toxicity. Moreover, some techniques such as single particle inductively coupled plasma mass spectrometry (single particle-ICP-MS) have been used to detect and quantify nanoparticles but are found to be laborious and are not suitable for some samples [202,203].

In addition, the existence of discrepancies between the results obtained for the same NMs and the important variety between them makes each NM unique, and extrapolating the results to others is very challenging. Despite various methods being available, there is still no broadly defined standard for assessing the toxicity of NMS in vitro and in vivo. Furthermore, the wide variety of NM characteristics can explain some discrepancies in the toxicity results. Changing just one parameter such as particle size can be enough to alter the toxicity. Moreover, other variable parameters such as exposure time, dosage, and cell lineage will also affect the results. Furthermore, it is important to know the proprieties of NMs such as the size, shape, capping agent, and speciation of the dissolved metal ions in the biological environment. It is also important to consider the production of artifacts in toxicity experiments. In general, multiple assays are based on optical detection by colorimetric and fluorimetric analyses. Thus, some NMs can interfere with these methodologies by changing parameters such as the adsorption capacity, optical properties, and hydrophobicity. In addition, some NMs can interfere with some cytotoxicity in vitro assays by scattering or absorbing light in the same spectral range of the test. Indeed, they can adsorb, deplete, and inactivate the reagents, or even interact with cell products like proteins [203,204,205,206]. To resolve these problems, it is necessary to make some adaptations including centrifugation, thorough washing, or even removing the supernatants. Furthermore, using more than one method and the appropriate controls helps to minimize artifacts or misinterpretations. To ensure more realistic and reproducible results, it is critical to define standard protocols for the minimum characteristics of NMs that must be studied such as size and shape, cells or organisms that will be used as well as the dosage and methods to assess the toxicity. It is also important to consider the application of nanomaterial transformations in the environment and study the chronic effects considering the exposure routes [206,207,208]. Recently, 3D-cellular culture has emerged as a possibility to assess toxicity under more realistic conditions considering the tissue architecture, complexity, development, and interactions. Despite this great potential, there are some questions to overcome regarding the complexity of the tissues, vascularization, and hypoxia in the cell culture [209,210,211]. In this regard, is also important to evaluate the effects of chronic exposure on living organisms, even at minimal doses and without apparent effects. Furthermore, it must be ensured that the studies apply adequate exposure times and doses to obtain information closer to reality.

Moreover, understanding the effects of NMs in complex and highly variable environments presents a great challenge. For example, evaluating all possible interactions of NMs in the atmosphere with a wide variety of contaminants, airflow, precipitation, seasonal variations, temperature, humidity, and others, remains a major challenge [212]. The characterization of NMs is also very complex, since many equipment such as electronic microscopy is expensive, time-consuming, and not very accessible to many laboratories, and different methodological approaches are needed to obtain more detailed information.

To assess the potential risks of NMs to the environment, it is important to identify the emission source from their fabrication to disposal. Mathematical models can be considered as an important tool to help in examining the risks and can be used in conjunction with the experimental data for regulatory assessment. However, the amount of data from the different stages of the life cycle of NMs are still scarce and more information is needed to refine these models and obtain more accurate results [213]. Another issue is the management of NMs throughout their useful life. During the fabrication, a small fraction can be released into the atmosphere [212,214]. Despite being a small fraction, the exposure through respiration and the transformations incurred in the air can be a significant route of toxicity.

Furthermore, in regions where NMs are produced or discarded, higher concentrations in the atmosphere are expected. Some strategies can be taken to reduce possible contamination including closed reactors and emission control systems during the synthesis [212]. Alternative synthesis methods such as using plant extracts and microorganisms can reduce the NMs’ toxicity, mainly the risks associated with their production and use [10,215]. Furthermore, as they do not need extreme temperatures, pH, and pressures, NMs may reduce the risks associated with the synthesis and production of by-products. As a result, an important reduction of waste, cost, and environmental impact. It has also been reported that green synthesis using vegetable extracts or oils tends to be simple and fast, requiring fewer steps and hence minimizing the possibility of exposure [31]. Safety and control measures also need to be taken during handling to prevent accidental NM emissions. Moreover, to reduce NM emissions in the workplace or outside, some approaches including containment barriers, ventilation, air purification, and air treatment can be applied [212].

Some NMs can be involved during the recycling process when associated with some products such as electronics, textiles, and construction materials, but this is poorly understood. Different processes are being developed to recover NMs from waste [216,217,218,219,220]. However, in general, recovery processes for metallic materials are often inefficient or nonexistent due to limits imposed by social behavior, cost, product design, recycling technologies, and the thermodynamics of separation.

Furthermore, in recent years, different countries have started to establish some laws to regulate the production, marketing, use, and disposal of NMs. The regulatory challenges are also enormous because NMs present large differences and a case-by-case assessment may not be efficient with the rapid growth of nanotechnology [221]. For example, concerns about respirable fibrous NMs have led many countries to create risk guidelines and establish risk exposure limits. In 2007, the British Standards Institution published a guideline to help understand and assess airborne exposure to NMs in the occupational environment and proposed an occupational exposure limit of 0.01 fibers/mL for fibrous NMs [222]. The Dutch Social and Economic Council (2012) recommended a similar occupational exposure limit for any fibrous NMs [223]. In 2013, the United States National Institute for Occupational Safety and Health (NIOSH) recommended an exposure limit for CNTs of 1 µg/m^3^ as an 8-h time-weighted average (TWA) of EC for the respirable range fraction [224]. Additionally, NIOSH recommends airborne exposure limits of 2.4 mg/m^3^ for fine TiO_2_ and 0.3 mg/m^3^ for ultrafine TiO_2_ as TWA concentrations for up to 10 h/day [225]. In Australia, substances containing 10% or more NMs need to be notified [226]. In 2017, the International Agency for Research on Cancer (IARC) considered MWCNTs are possibly carcinogenic to humans, while SWCNTs and others were not [227].

Despite these restrictions, there are already a variety of products with authorized production and sale. In 2012, the FDA approved the first clinical trial using NMs. The FDA approved the first clinical trials aiming to use AuNPs for photothermal therapy against lung cancer. European Food Safety Authority authorized TiO_2_ as a food additive (Annex II of Regulation (EC) No. 1333/2008), but in 2021, TiO_2_ was no longer considered safe as a food additive. France was the first European county to ban this use for TiO_2_ [228]. In the USA, the FDA allows the use of TiO_2_ as a food color if the quantity of TiO_2_ does not exceed 1 percent by weight of the food.

For a more adequate and standardized assessment of the risks associated with NMs, many countries and institutions have created guidelines and protocols to facilitate these analyses. The ISO/TS 12901 guides occupational health and safety measures relating to NMs including personal protection and the management of accidental releases. Furthermore, collaborative projects such as NanoReg and NanoValid have been developed to support regulatory agencies and establish protocols for the characterization and evaluation of the toxicity of NMs [229].

It is important to highlight that nanotechnology legislation is essential to ensure the quality and safety of nanoproducts. Standardization regarding metrological and nanotoxicological parameters is essential. The International Organization for Standardization has established standards for nanotechnology with ISO/TC-229 to analyze nanotechnological aspects related to health and the environment, among other ISO standards under development [230]. Similarly, the Food and Drug Administration (FDA) has maintained its regulatory policy regarding nanotechnology. It has already developed guidelines for safe and efficient nanotechnological processes concerning human, animal, and environmental health. The monitoring of commercialized products composed of NMs, for example, is carried out continuously for the protection of consumers.

## 7. Conclusions

Nanotechnology has enabled tremendous advances in various fields of science. Further developments are necessary to improve the existing NMs while discovering novel ones that promise benefits to different technological fields. However, as these NMs are unique with significant differences, extensive studies are needed to better understand the real risks and limitations of using NMs. Nevertheless, there still exists some discrepancies in the reported results regarding the risks associated with NMs besides the lack of comparisons associated with the standardization limits. In-depth monitoring from the synthesis to the destination will certainly allow the risks to be assessed and minimized at each stage. Green synthesis can be an important alternative for the reduction in risks. It is crucial to invest in eco-friendly fabrication methods and ensure that proper and sustainable disposal is adopted. Furthermore, the implementation of 3D cell culture can be an auspicious tool in the initial assessment of the toxicity of a nanomaterial. Broader studies that consider different environmental conditions such as high and low temperatures are fundamental in evaluating the stability of these nanostructures in a natural environment. Additionally, evaluating the timeframe in which products containing NMs maintain quality is essential, ensuring that they are only marketed within a minimum quality standard.

**Figure 7:**
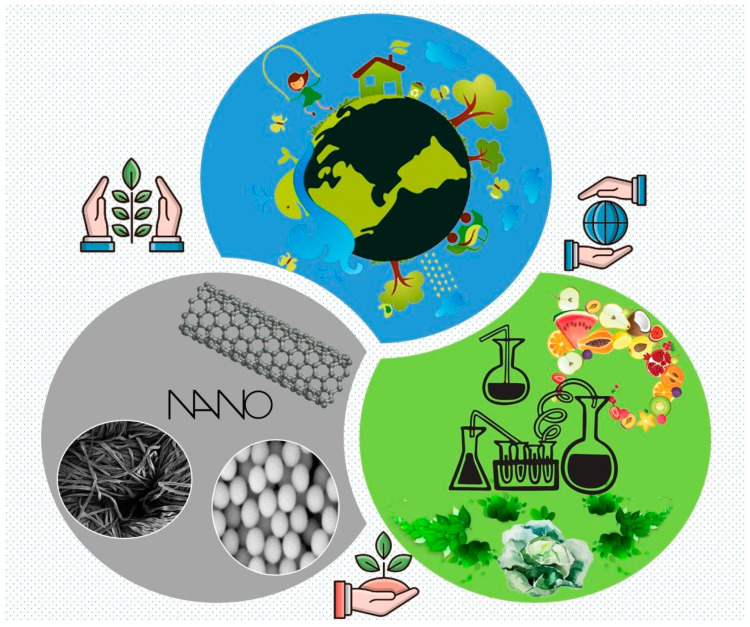
Nanotechnology can be a powerful development tool for society while considering sustainability as an important factor in this process and using more eco-friendly approaches during production.

In summary, to assess the potential risks of NMs on the environment and biological systems, it is discerning to perform in-depth and breadth characterizations of NMs and examine the potential transformations and consequences on the bioavailability and toxicity. It is also essential to assess the routes and duration of exposure, stability, and interaction with other contaminants or by-products as well as carry out bioassays that provide more practical information. Nevertheless, it is important to highlight that nanotechnology can benefit society and economic development (Figure 7).

## Figures and Tables

**Table 1:** Examples of different applications of nanomaterials involving biological systems.

Applications	Functions	Examples	References
Antimicrobial	Combats bacteria, viruses, fungi, and parasites.	NPs (mainly AgNPs) and graphene	[3,53,54,55,56,57]
Nanosensor	Detection of pathogens and molecules Monitoring of environmental conditions	NPs, carbon nanotubes (CNTs), quantum dots and graphene	[58,59,60,61]
Remediation	Adsorption and degradation of organic and inorganic pollutants	NPs, carbon nanotubes (CNTs), graphene, and nanocomposites	[1,62,63,64,65]
Cosmetics	Ultraviolet (UV) radiation protection	TiO_2_ and ZnONPs	[5,6,11]
Drug delivery	Controlled release of the drug Improvement of characteristics such as stability and solubility, biocompatibility	NPs and Graphene	[66,67,68]
Antitumor	Acts in the tumor microenvironment Induce cell death Photocatalytic properties	Graphene, CNTs, and NPs	[8,69,70,71,72,73,74,75,76,77,78,79,80,81]
Regenerative medicine	Scaffolds Induces cell differentiation and proliferation Modulates inflammatory response	Graphene and NPs	[82,83,84,85]
Agriculture	Increase agricultural productivity Pesticide Fertilizers	NPs, CNT, graphene, and quantum dots	[86,87,88]
